# Preoperative chemoradiation with capecitabine, irinotecan and cetuximab in rectal cancer: significance of pre-treatment and post-resection RAS mutations

**DOI:** 10.1038/bjc.2017.294

**Published:** 2017-08-31

**Authors:** Simon Gollins, Nick West, David Sebag-Montefiore, Arthur Sun Myint, Mark Saunders, Shabbir Susnerwala, Phil Quirke, Sharadah Essapen, Leslie Samuel, Bruce Sizer, Jane Worlding, Katie Southward, Gemma Hemmings, Emma Tinkler-Hundal, Morag Taylor, Daniel Bottomley, Philip Chambers, Emma Lawrie, Andre Lopes, Sandy Beare

**Affiliations:** 1 Department of Oncology, North Wales Cancer Treatment Centre, Bodelwyddan, Denbighshire LL18 5UJ, UK; 2 Leeds Institute of Cancer and Pathology, University of Leeds, Leeds LS9 7TF, UK; 3 St James’ Institute of Oncology, University of Leeds, Leeds LS9 7TF, UK; 4 Clatterbridge Cancer Centre, Clatterbridge Road, Wirral CH63 4JY, UK; 5 The Christie NHS Foundation Trust, Withington, Manchester M20 4BX, UK; 6 Royal Preston Hospital, Fulwood, Preston PR2 9HT, UK; 7 Pathology and Tumour Biology, Level 4 Wellcome Trust Brenner Building, St James University Hospital, Beckett Street, Leeds LS9 7TF, UK; 8 St Luke’s Cancer Centre, Egerton Road, Guildford GU2 7XX, UK; 9 Aberdeen Royal Infirmary, Foresterhill, Aberdeen AB25 2ZN, UK; 10 Colchester General Hospital, Turner Road, Colchester CO4 5JL, UK; 11 University Hospitals Coventry and Warwickshire NHS Trust, Clifford Bridge Road, Coventry CV2 2DX, UK; 12 Cancer Research UK & UCL Cancer Trials Centre, University College London, 90 Tottenham Court Road, London W1T 4TJ, UK

**Keywords:** locally advanced rectal cancer, cetuximab-containing chemoradiation, *RAS* mutations, intra-tumoural clonal heterogeneity, treatment response, next generation sequencing

## Abstract

**Background:**

The influence of EGFR pathway mutations on cetuximab-containing rectal cancer preoperative chemoradiation (CRT) is uncertain.

**Methods:**

In a prospective phase II trial (EXCITE), patients with magnetic resonance imaging (MRI)-defined non-metastatic rectal adenocarinoma threatening/involving the surgical resection plane received pelvic radiotherapy with concurrent capecitabine, irinotecan and cetuximab. Resection was recommended 8 weeks later. The primary endpoint was histopathologically clear (R0) resection margin. Pre-planned retrospective DNA pyrosequencing (PS) and next generation sequencing (NGS) of *KRAS*, *NRAS*, *PIK3CA* and *BRAF* was performed on the pre-treatment biopsy and resected specimen.

**Results:**

Eighty-two patients were recruited and 76 underwent surgery, with R0 resection in 67 (82%, 90%CI: 73–88%) (four patients with clinical complete response declined surgery). Twenty–four patients (30%) had an excellent clinical or pathological response (ECPR). Using NGS 24 (46%) of 52 matched biopsies/resections were discrepant: ten patients (19%) gained 13 new resection mutations compared to biopsy (12 *KRAS*, one *PIK3CA*) and 18 (35%) lost 22 mutations (15 *KRAS*, 7 *PIK3CA*). Tumours only ever testing *RAS* wild-type had significantly greater ECPR than tumours with either biopsy or resection *RAS* mutations (14/29 [48%] *vs* 10/51 [20%], *P*=0.008), with a trend towards increased overall survival (HR 0.23, 95% CI 0.05–1.03, *P*=0.055).

**Conclusions:**

This regimen was feasible and the primary study endpoint was met. For the first time using pre-operative rectal CRT, emergence of clinically important new resection mutations is described, likely reflecting intratumoural heterogeneity manifesting either as treatment-driven selective clonal expansion or a geographical biopsy sampling miss.

Preoperative chemoradiation (CRT) is a standard treatment in locally advanced rectal cancer (Gerard *et al*, 2006; National Institute for Health and Care Excellence, 2011; Schmoll *et al*, 2012; Bossett *et al*, 2014) using a concurrent fluoropyrimidine during 5 weeks of pelvic radiotherapy. To increase efficacy, adding a second drug has been investigated. The combination of capecitabine and irinotecan has been studied in phase II trials, including by our own group (Gollins *et al*, 2011) with promising response and survival.

The epidermal growth factor receptor (EGFR) is over-expressed in approximately 60% of rectal cancers and associated with worse prognosis (Giralt *et al*, 2005). Cetuximab is an anti-EGFR chimeric monoclonal antibody demonstrating benefit when added to chemotherapy for metastatic colorectal cancer (mCRC) (Cunningham *et al*, 2004; Van Cutsem *et al*, 2009) but is ineffective in the presence of *RAS*-activating mutations (Van Cutsem *et al*, 2015).

Preclinical data indicated that cetuximab is a radiation sensitiser and in head and neck cancer cetuximab combined with radiotherapy improved median overall survival (OS) (Bonner *et al*, 2006). However, the benefit of cetuximab in addition to concurrent single or doublet chemotherapy in rectal cancer CRT remains uncertain. No phase III studies have been reported although in early phase trials pathological complete response (pCR) rates appear no greater than previously reported using CRT without cetuximab, even when tumours were divided into *KRAS* wild-type *vs* mutated (Clancy *et al*, 2013; Greenhalgh *et al*, 2016). However, a randomised phase II trial (EXPERT-C) used 12 weeks of oxaliplatin/capecitabine chemotherapy followed by CRT with concurrent capecitabine, then surgery, then 12 further weeks of oxaliplatin/capecitabine or the same regime plus weekly cetuximab. In a subset of 90 KRAS/BRAF wild-type patients there was a suggested improvement in overall response rate and survival with cetuximab (Dewdney *et al*, 2012).

Our previous RICE study included 110 patients with similar magnetic resonance imaging (MRI)-defined entry criteria to the current study (EXCITE), examining CRT including irinotecan and capecitabine without cetuximab (Gollins *et al*, 2011). EXCITE assessed the toxicity, compliance and effectiveness of adding cetuximab to the doublet of capecitabine/irinotecan during CRT.

RICE delivered capecitabine 7 days per week throughout CRT whereas EXCITE gave capecitabine at similar dose 5 days per week with radiotherapy, to avoid excessive toxicity. In EXCITE a pre-planned retrospective analysis was carried out of EGFR pathway mutations, using pyrosequencing (PS) and next generation sequencing (NGS) of pre-treatment biopsy and post-resection specimen, examining their influence on response and survival.

## Materials and methods

### Eligibility

EXCITE (EUDRACT 2007-006701-25) was a UK multicentre, open-label, single arm phase II trial (full protocol available at http://www.ctc.ucl.ac.uk/TrialDetails.aspx?Trial=76&TherA=7). Eligible adult patients of World Health Organisation Performance Status 0–1 had histopathologically confirmed rectal adenocarcinoma with distal limit ⩽12 cm from anal verge using rigid sigmoidoscopy. Pelvic MRI-defined inclusion criteria comprised mesorectal fascia (MRF) being threatened (tumour ⩽1 mm from MRF), involved or breached, or low tumours <5 cm from the anal verge. CT chest and abdomen excluded metastatic disease and haematological and biochemical indices were satisfactory. Patients were deemed fit to receive all study treatments.

### Treatment

A CT-planned pelvic volume received megavoltage radiotherapy at 45 Gy in 25 daily fractions of 1.8 Gy treating 5 days per week Monday–Friday. Patients received oral capecitabine 650 mg m^−2^ b.d. on the days of radiotherapy only, cetuximab 400 mg m^−2^ intravenously (i.v.) 1 week prior to radiotherapy then 250 mg m^−2^ once-weekly during weeks 1–5 of radiotherapy and irinotecan 60 mg m^−2^ i.v. once-weekly during weeks 1–4 of radiotherapy.

Surgery was recommended at 8 weeks following CRT. Post-surgery, adjuvant chemotherapy was given at the treating physician’s discretion. Patients were followed for 3 years post-surgery to assess progression, survival and post-surgical and long-term morbidity.

### Assessments

The primary outcome measure was R0 resection rate. Secondary outcomes were treatment compliance, grade 3 or 4 toxicity (NCI CTCAE version 3.0), post-operative morbidity, pathological response, progression-free survival (PFS) and OS.

R0 resection was defined as histologically clear margins >1 mm, R1 microscopically involved margins ⩽1 mm and R2 macroscopically involved margins. Histological tumour regression grade (TRG) was scored by the local pathologist as 0 (no regression), 1 (dominant tumour mass, <25% fibrosis), 2 (26–50% fibrosis), 3 (dominant fibrosis, >50% tumour regression), 4 (‘microfoci’: scattered single tumour cells only) and 5 (pCR: no residual viable carcinoma on extensive examination of the resected specimen), based on Rödel *et al*, 2005 with additional TRG 4 based on our previous work, wherein we showed that patients with either a pCR (TRG5) or microfoci (TRG4) following CRT, had excellent long-term survival outcome compared to all other patients achieving lesser degrees of downstaging (Gollins *et al*, 2011).

Pre-treatment biopsy and surgical resection formalin-fixed paraffin-embedded tumour tissue was collected and DNA extracted at the Pathology and Tumour Biology laboratory, University of Leeds. EGFR signalling pathway mutations were analysed post-trial including *KRAS* codons 12, 13, 61, 146, *NRAS* codons 12, 13, 61, *PIK3CA* codons 542, 545, 546, 1047, and the *BRAF* V600E hotspot. Pyrosequencing (Richman *et al*, 2009) and NGS (Supplementary Online Material) were performed by the laboratory on the same specimen.

Mutated DNA was scored as present if it constituted at least 5% of the total DNA analysed. The 5% cut-off was chosen after testing a series of known dilutions to ascertain what could reliably be detected without interference from false positives. The main analysis examined *KRAS* and *NRAS* mutations in keeping with subsequent evidence that both *KRAS* and *NRAS* mutations reduce cetuximab effectiveness in mCRC (Van Cutsem *et al*, 2015), reflected in the current product licence.

### Statistical analysis

The primary endpoint of R0 resection rate with single agent fluropyrimidine CRT was estimated at 55% and adding irinotecan and cetuximab were expected to increase this to at least 75%. Using a Fleming’s design with 80% power and one-sided 5% level test of statistical significance, 35 patients would be required. The initial recruitment target was therefore 40 patients, allowing for drop-outs. As recruitment commenced in April 2009, evidence emerged in the first line metastatic CRYSTAL trial (also published in April 2009), suggesting that cetuximab was beneficial in *KRAS* wild-type but not *KRAS*-mutated tumours (Van Cutsem *et al*, 2009). However, it was unknown whether this would apply using cetuximab concurrently with CRT. The sample size was increased to 80 patients to give a 97% chance of at least 40 *KRAS* wild-type tumours for R0 resection rate analysis, as mutated *KRAS* was expected in 35–40% of colorectal adenocarcinomas. The protocol-specified, pre-planned intention was to compare outcomes for *RAS* wild-type *vs* mutant patients. This biomarker analysis was exploratory, to assess the association with resection and regression status and time to event endpoints.

Data were analysed with the Stata SE 14 statistical package according to intention to treat. Toxicity analyses were conducted only in those patients who commenced treatment and the surgical complications analysis only in those who had surgery.

Proportions were compared using chi-square tests (Fishers Exact Test where appropriate). Kaplan–Meier censored survival curves were used to present survival data with log-rank *P-*values. Survival was calculated from the date of trial registration. PFS was the time to the first event of local pelvic recurrence, distant metastases, or death, and OS to death. Hazard ratios (HR) were derived from Cox regression analysis. Pearson _X^2^ test of independence to two-sided significance was used where indicated.

The trial was approved by National Research Ethics Service Committee: South Central–Oxford B (08/H0605/6), the Medicines and Healthcare products Regulatory Agency (Clinical Trial Authorisation number 20363/0228/001-0001), and by each participating NHS Trust’s Research and Development department. Informed consent was obtained from all patients.

## Results

Patients were recruited between April 2009 and October 2011 from nine UK radiotherapy centres. Pre-treatment characteristics are shown in Table 1, confirming locally advanced cancers, with 39 (48%) involving or breaching the MRF and the remainder margin-threatened.

One poor-performance status patient did not start treatment. Another received the initial cetuximab dose only and was then withdrawn from the trial by the treating clinician, who considered the radiotherapy treatment volume too large. As the primary endpoint was histologically determined post-surgery, they were replaced with two additional patients. Intention-to-treat analysis included all 82 patients where appropriate.

Most patients received the full dose of radiotherapy (76 patients, 93%), irinotecan (56 patients, 68%) and cetuximab (60 patients, 73%) but only 39 (48%) received the full capecitabine dose (Supplementary Online Material Table 1).

The commonest serious adverse events during CRT were grade 3 diarrhoea, acneiform rash and fatigue (Table 2). Five of six non-haematological grade 4 adverse events were thromboembolic. There were no treatment-related deaths prior to surgery.

Of the 80 patients commencing radiotherapy, 76 underwent surgery, a median of 72 days (inter-quartile range (IQR) 62–94.5 days) post CRT completion with half undergoing an abdominoperineal excision (Supplementary Online Material Table 2). Contrary to protocol, four patients with an endoscopically- and MRI-confirmed complete clinical response (cCR) declined surgery and were managed with a ‘watch-and-wait’ deferral of surgery strategy off-trial. One postoperative death occurred within 30 days of surgery from bowel obstruction (Supplementary Online Material Table 2).

Post-surgery, 54 patients (71%) received adjuvant chemotherapy, 23 using a fluoropyrimidine (fluorouracil or capecitabine), 28 a fluoropyrimidine/oxaliplatin combination and 3 other.

A negative (R0) resection margin was achieved in 67 out of 82 patients: (82%, 90% CI: 73–88%), thereby meeting the primary endpoint (lower 90% CI bound excluded 55%). A pCR (ypT0ypN0; TRG 5) was found in 14 patients (17%) and near-complete (microfoci; TRG 4) in 6 (7%) (Table 3).

We previously showed that rectal CRT patients achieving TRG 4–5 had superior survival to other patients (Gollins *et al*, 2011). Management of four EXCITE patients with cCR by ‘watch-and-wait’ was unexpected but they were included with resected patients with TRG 4–5 for survival analysis; therefore, 24 of 80 patients who commenced radiotherapy (30%) had an excellent clinical or pathological response (ECPR).

The median follow-up was 37.4 months (IQR: 26.8–38.9 months). One patient developed local pelvic recurrence only, 15 distant metastases only and four both local and distant relapse. Fifteen patients died and 26 had a PFS event. The four cCR patients managed conservatively remained disease-free at 24, 39, 42 and 42 months. Thirty-six-month PFS for all 82 recruited patients was 67% (95% CI:55–76%) and OS 80% (95% CI:69–87%).

Twenty-four of the 56 (43%) non-ECPR patients either progressed or died compared to 2 of 24 (8%) with ECPR. The 36-month PFS for non-ECPR patients was 54% (95% CI: 39–66%) and for ECPR 95% (95% CI: 74–99%) and OS 73% (95% CI: 58–83%) *vs* 95% (95% CI: 72–99%) (Supplementary Online Material Figure 1a and b).

### EGFR pathway mutation status

Mutation status was retrospectively determined on biopsy samples from 78 patients and resection specimens from 54, with 52 matched biopsy/resection samples (Table 4). Resection mutation status could not be determined in the 24 patients with ECPR because of no or very little viable residual cancer.

### Biopsy samples

Using PS, 45 (58%) of 78 biopsy samples had at least one EGFR pathway mutation (52 mutations total), the majority in *KRAS* codon 12 (Table 4). next generation sequencing was more sensitive, identifying a further 21 mutations, the majority in *KRAS*, all but one at a mutational percentage of 5–10% of the total DNA present. By PS 37 (47%) and by NGS 44 (56%) of biopsies were *RAS* (*KRAS* or *NRAS*) mutated.

By NGS 33 of 78 biopsy samples had a single, 12 a double, 4 a triple and one a quadruple mutation (Supplementary Online Material Table 3).

### Resection samples

Using PS, 32 (59%) of 54 resection samples had at least one EGFR mutation (35 mutations total), with an additional 8 identified by NGS (Table 4). One PS mutation was not confirmed with NGS. By PS/NGS, 33 resections (61%) were *RAS* mutated. Twenty-six resections had a single, 7 a double and one a triple mutation (Supplementary Online Material Table 3).

### Matched biopsy/resection samples

In the 52 patients with matched biopsy/resection specimens, 24 patients (46%) showed a discrepancy between biopsy and resection (Table 5).

Ten patients (19%) gained 13 new resection mutations compared to biopsy (10 *KRAS* 12, two *KRAS* 146 and one *PIK3CA*). Nine patients gained at least one new *RAS* mutation and five of these changed their overall *RAS* mutation status from biopsy wild-type to resection mutated. Most new *KRAS* mutations (9 of 12) were present above 20% of the total DNA analysed.

Eighteen patients (35%) lost 22 mutations between biopsy and resection (three *KRAS* 12, six *KRAS* 13, six *KRAS* 146, seven *PIK3CA*). In the 14 patients solely losing mutations, five could be detected at <5% in the resection specimen (KRAS 13 at 1% in three patients and 4% in one and KRAS 146 at 2% in one).

Four of the above patients both lost and gained mutations.

### The relationship between RAS mutation status and histological response and survival

*RAS* mutation status was not related to R0 resection rate (Table 6). The difference in ECPR rate between biopsy *RAS* mutated *vs* biopsy wild-type tumours was not significant (23% *vs* 41% respectively, *P*=0.090). However, there was evidence that the new resection RAS mutations were clinically important, in that patients who were ‘anytime’ *RAS* mutated in either biopsy or resection had a lower ECPR rate (10/51: 20%) compared to those who only ever tested *RAS* wild-type (14/29: 48%, *P*=0.008).

**Figure 1:**
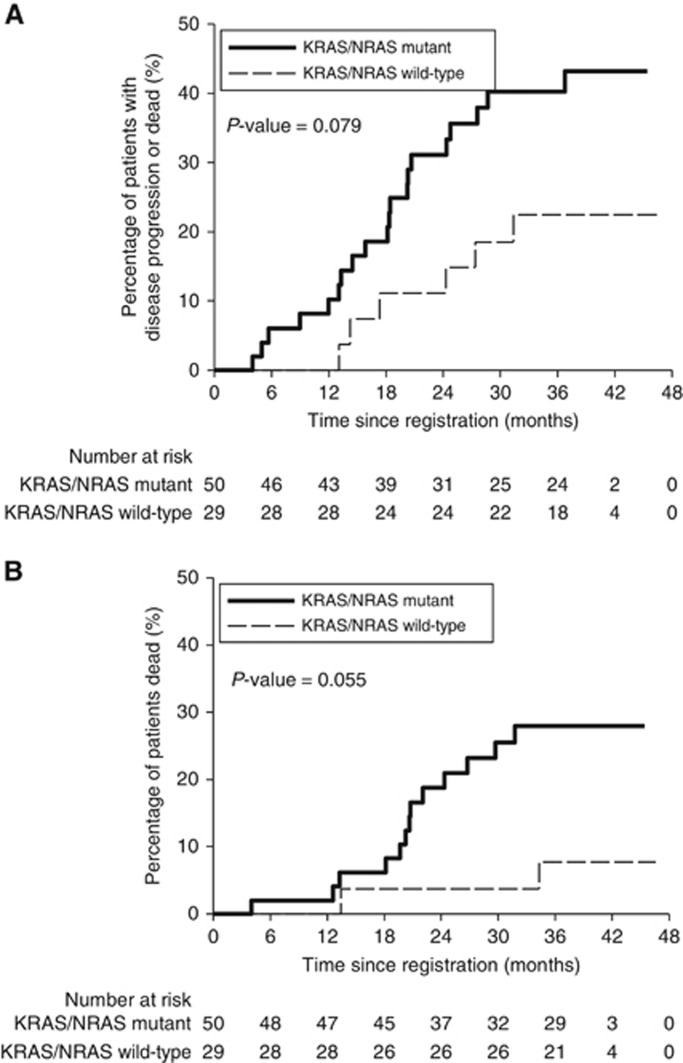
**Relationship between RAS mutation status and progression-free and overall survival.** (**A**) progression-free survival and (**B**) overall survival in patients who were *RAS* mutated in either pre-treatment biopsy or resected specimen (‘anytime mutant’) versus patients whose specimens only ever tested *RAS* wild-type.

There was some evidence of an improvement in PFS (HR 0.44 (95% CI: 0.18–1.10), *P*=0.079) and OS (HR 0.23 (95% CI: 0.05–1.03), *P*=0.055) for wild-type compared to anytime-mutated cancers (Figure 1A and B, Table 6), although this did not reach statistical significance at the 5% level.

## Discussion

The regimen investigated was feasible, with acceptable rates of treatment-related toxicity. EXCITE met its primary R0 resection rate end point, although this was not improved compared to our previous study (RICE) using concurrent irinotecan and capecitabine without cetuximab (82% *vs* 89% respectively) (Gollins *et al*, 2011). Likewise the EXCITE overall pCR (TRG 5) rate was similar (EXCITE 14/82: 17% *vs* RICE 24/110: 22%), as was 3-year PFS (EXCITE 67% and RICE 64%). In this respect our study was similar to other early phase trials using concurrent cetuximab, which have broadly failed to demonstrate an increase in pCR rate compared to historical series using chemotherapy alone (Clancy *et al*, 2013 and Greenhalgh *et al*, 2016).

Despite delivery of capecitabine at 650 mg m^−2^ b.d. 5 days per week compared to 7 in RICE, less than half our patients received the full capecitabine dose. Two previous studies have examined capecitabine/irinotecan/cetuximab concurrent with CRT (Erben *et al*, 2011; Kim *et al*, 2013). One of these reported high compliance with a lower capecitabine dose of 400–500 mg m^−2^ (Erben *et al*, 2011) although this dose is significantly lower than when using capecitabine alone (typically 825 mg m^−2^). Theoretically such lower achievable dose intensity may be due to increased toxicity from the addition of cetuximab, as suggested for other tumour sites (Crosby *et al*, 2013). In the current study capecitabine dose reductions were protocol-driven. In the presence of grade 2 diarrhoea (the most common toxicity), capecitabine dose was to be reduced to 75% ‘if no response to loperamide’. No time course over which to make this assessment was recommended in the protocol, however, which may have led to an increased tendency for capecitabine dose reduction compared to irinotecan, where the protocol stated that irinotecan dose was only to be lowered if there was grade 3 toxicity.

Five patients in EXCITE experienced grade 4 thromboembolism, which is greater than RICE (0%). The reason for this difference is unclear although in no patient did this cause death or compromise surgery. The two previous studies examining the combination of irinotecan/capecitabine/cetuximab did not record any thromboembolism associated with the regimen (Erben *et al*, 2011; Kim *et al*, 2013).

Unique features of the current study firstly included access to the full set of biopsy and resection specimens for analysis of mutational status. Secondly, in contrast to previously reported studies we used the sensitive methodology of NGS for mutation analysis. Thirdly, we studied an MRI-defined group of locally advanced cancers whose mutational burden may be greater than earlier stage disease.

In a substantial proportion of patients (46%) we found a discrepancy in EGFR pathway mutations (mainly in *KRAS*) comparing rectal cancer tissue pre- and post-CRT, which to our knowledge has not previously been described. Even using NGS, only one of the 12 new resection *KRAS* mutations could be detected at <5% in the corresponding original biopsy (*KRAS* 12 at 1%).

In the 9 patients in which emergent new *RAS* mutations were identified in the resected specimen, these appeared to be clinically important in being associated with worse response and survival. Our findings agree with previous reports in this context in that if solely biopsy RAS mutations are considered, we did not find a statistically significant decrease in EPCR rate compared to wild-type. However, when the resection mutation status was additionally taken into account (‘anytime’ mutated *vs* wild type), the difference in response was significantly increased for wild-type, with a trend towards improved survival. The implication is that clinically important low-level *RAS* mutations in the pre-treatment biopsy, that contribute to reduced response, are not identifiable with current biopsy and sequencing techniques, even as sensitive as NGS, representing a potential challenge to personalised medicine.

Possible explanations for emergence of new mutations in the resection are either the treatment-driven selection and expansion of initially undetectable low-level clones, biopsies which geographically missed the particular region of the tumour containing a mutation, or both. Our findings thus provide evidence for intratumoural clonal heterogeneity (ICH) in rectal cancer, which lies at the root of either explanation. In the current study we found additional evidence for ICH with a *KRAS* mutation being found concurrent with another *KRAS* or *NRAS* or *BRAF* mutation in eight biopsies, which is unusual (De Rook *et al*, 2010).

Disappearance of mutations in the current study could be related to CRT response. Following macrodissection there was sufficient neoplastic cell content (mean 25%) in the 14 resected tumours solely losing mutations, to have detected the original biopsy mutations if present.

There is increasing awareness of ICH in colorectal cancer, with potential clinical relevance. A study sampling multiple locations from the same colorectal tumour showed that using PS, 7 of 69 primary tumours (10%) demonstrated ICH (Richman *et al*, 2011) and genomic profiles of primary tumours and metastases are not always concordant (Vogelstein *et al*, 2013). On analysing 349 individual lymph glands from 15 colorectal tumours, uniformly high ICH and subclone mixing was demonstrated in both primary tumour and lymph nodes, and it was proposed that most detectable ICH results from early subclonal alterations (Sottoriva *et al*, 2015). During treatment with anti-EGFR monoclonal antibodies, emergence of *RAS* and other mutations can be identified in circulating tumour DNA (ctDNA) before clinically apparent disease progression (Diaz *et al* 2012; Siravegna *et al* 2015). Using RNA transcriptomic analysis, it has recently been shown that patients can be simultaneously classified into multiple diagnostically relevant subgroups based purely on the tumoural region analysed (Dunne *et al*, 2016).

In the current study it does seem likely that at least some of the emergent resection mutations that were not identified in pre-treatment biopsy arose because of treatment-driven clonal expansion, because most newly identified *KRAS* mutations (9 of 12) were present above 20% of the total DNA analysed. By definition, these mutations have arisen within an approximate 3-month period from biopsy to resection, implying that rapid clonal selection may have occurred, possibly accelerated by the well-known phenomenon of radiotherapy-induced accelerated repopulation (Willers and Held, 2006).

A limitation of the current study is the relatively small sample size and non-randomised nature, meaning that these observations remain hypothesis-generating and no conclusions can be drawn for use in routine clinical practice. It is not known if a similar emergence of resection *RAS* mutations would occur if patients were treated with CRT containing irinotecan and capecitabine alone without cetuximab. We are currently examining our previous RICE trial from this point of view.

In terms of future research, there are currently no recommended national standards for pre-treatment rectal biopsy in routine clinical practice. There is a need to define biopsy standards in terms of number of biopsies, volume and location, in order to increase the pre-treatment sensitivity of identifying clinically relevant mutations if preset. The use of NGS will also maximise sensitivity for identifying such mutations. In addition, sequential biopsy of the primary tumour during treatment may allow more early definition of emerging mutations, which could influence treatment approach. The use of liquid biopsies taken at baseline and at intervals during treatment may give information on such emergent mutational changes, without the need for repeat tissue biopsy (Spindler *et al*, 2015).

In summary, the regimen studied was feasible and met its primary R0 resection endpoint. Using the sensitive technology of NGS, comparing biopsy with resection, we describe for the first time substantial loss and gain of EGFR pathway mutations (mainly *KRAS*) in locally advanced rectal cancer undergoing pre-operative CRT. Appearance of new, initially undetectable *RAS* mutations, was related to significantly decreased response and a trend to inferior survival in tumours that were RAS mutated in either biopsy or resection compared to those only ever testing wild-type. Failure to detect such clinically important emergent resection mutations in pre-treatment biopsies may be related to a lack of influence of RAS mutation status on response in previous reports. This phenomenon is likely to be due to ICH manifesting as either treatment-driven selection of mutated clones or a biopsy geographical miss, thereby presenting a challenge to personalised medicine. Our findings highlight an urgent need to define a minimum standard for adequate pre-treatment biopsies in routine clinical practice.

## Figures and Tables

**Table 1:** Baseline characteristics of patients in EXCITE

**Baseline characteristics**	**Number of patients (%)**
**Gender**	
Female	21 (26%)
Male	61 (74%)
**Age at registration (years)**	
Median (range)	62 (26–79) [*n*=82]
**WHO performance status**	
0	62 (76%)
1	20 (24%)
**Defunctioning stoma**	
Ileostomy	3 (4%)
Colostomy	4 (5%)
None	75 (91%)
**Distance of distal end of tumour from anal verge using rigid sigmoidoscopy (mm)^a^**	
Median (range)	50 (0–130) [*n*=63]
**Distance of distal end of tumour from anal verge using MRI (mm)^a^**	
Median (range)	50 (0–120) [*n*=78]
**Maximum superior-inferior tumour dimension (mm)**	
Median (range)	51 (5–110) [*n*=76]
**Maximum tumour diameter in a plane perpendicular to the longitudinal central axis of the rectum (mm)**	
Median (range)	28 (10–100) [*n*=55]
Not measurable [*n*=25]	
Missing [*n*=2]	
**mrT-stage**	
T2	6 (7%)
T3	67 (82%)
T4	9 (11%)
**mrN-stage**	
N0	14 (17%)
N1	42 (51%)
N2	26 (32%)
**M-stage**	
M0	82 (100%)
**Mesorectal edge on MRI scan**	
Potentially involved (⩽ 1mm gap)	43 (52%)
Involved, not breached	22 (27%)
Breached	17 (21%)
Total	82 (100%)

Abbreviation: MRI=magnetic resonance imaging.

a All 82 patients had a measurement for distance of distal end of tumour from anal verge using either rigid sigmoidoscopy or MRI.

**Table 2:** Grade 3–4 adverse events occurring during and up to 4 weeks following completion of CRT (based on 81 patients that had some treatment)

	**Grade 3**	**Grade 4**
	**No. of patients (%)**	**No. of patients (%)**
**Haematological adverse events**		
Anaemia	1 (1%)	1 (1%)
Leucopoenia	5 (6%)	1 (1%)
Thrombocytopenia	0 (0%)	1 (1%)
Neutropenia	4 (5%)	1 (1%)
Febrile neutropenia	1 (1%)	1 (1%)
Any haematological AE	10 (12%)	4 (5%)
**Non-haematological adverse events**		
Diarrhoea	20 (25%)	0 (0%)
Acneiform rash	7 (9%)	0 (0%)
Fatigue	6 (8%)	0 (0%)
Dehydration	1 (1%)	0 (0%)
Pyrexia/Fever	1 (1%)	0 (0%)
Headache	1 (1%)	0 (0%)
Insomnia	1 (1%)	0 (0%)
Taste disturbance	1 (1%)	0 (0%)
Nausea	1 (1%)	0 (0%)
Vomiting	1 (1%)	0 (0%)
Urticaria	1 (1%)	0 (0%)
Other rash/skin reactions^a^	7 (9%)	0 (0%)
Anal/rectal/bowel complications^b^	6 (7%)	0 (0%)
Thrombotic event^c^	1 (1%)	5 (6%)
Other^d^	4 (5%)	1 (1%)
Any non-haematological adverse event	36 (44%)	6 (7%)
Any adverse event	38 (47%)	10 (12%)

Abbreviations: AE=adverse event; CRT=chemoradiation.

a Skin related toxicity (2); rash (1); radiotherapy skin reaction (1); papular rash (1); shingles (1); perineal desquamation (1).

b Rectal pain (1); bowel obstruction (1); tenesmus (1); sore anal verge (1); pain passing stools/rectal pain (1); perianal abscess (1).

c Grade 3: deep vein thrombosis (1). Grade 4: pulmonary embolism (3); thrombosis/embolism (2).

d Grade 3: pulmonary infection(1); vasovagal attack(1); urinary tract problems (1); dry cracked heels (1). Grade 4: urinary tract infection (1).

**Table 3:** Histology of resected cancers^a^

**Confirmed resection status**	**Number (%)**
R0	67 (82%)
R1	8 (10%)
R2	1 (1%)
Did not have surgery	6 (7%)
**Tumour regression grade**	**Number (%)**
Grade 0	10 (12%)
Grade 1	11 (13%)
Grade 2	18 (22%)
Grade 3	17 (21%)
Grade 4	6 (7%)
Grade 5: pCR	14 (17%)
Did not have surgery	6 (7%)
**T stage**	**Number (%)**
ypT0	14 (17%)
ypT1	1 (1%)
ypT2	17 (21%)
ypT3	40 (49%)
ypT4	3 (4%)
ypTx	1 (1%)
Did not have surgery	6 (7%)
**N stage**	**Number (%)**
ypN0	52 (63%)
ypN1	15 (18%)
ypN2	9 (11%)
Did not have surgery	6 (7%)
**T stage of resected specimen compared to pre-treatment MRI scan**	**Number (%)**
Downstaged	37 (49%)
Unchanged	33 (44%)
Upstaged	5 (7%)
Total	75 (100%)
**N stage of resected specimen compared to pre-treatment MRI scan**	**Number (%)**
N1-2 downstaged	50 (78%)
N1-2 unchanged	12 (19%)
N1-2 upstaged	2 (3%)
Total	64 (100%)
N0 unchanged	9 (75%)
N0 upstaged	3 (25%)
Total	12 (100%)

Abbreviations: CRT=chemoradiation; MRI=magnetic resonance imaging.

a 82 patients were recruited to EXCITE in total and six patients did not have surgery because two patients did not commence CRT and in 4 patients a ‘wait and watch’ approach was adopted by the treating team because of a complete clinical response to CRT.

**Table 4:** Mutations detected in biopsy and resection samples by PS and NGS

**Mutation**	**Absolute number of mutations detected by PS**	**Detail**	**Absolute number of** ***additional*** **mutations detected by NGS**	**Detail (percentage mutated DNA)**
**Biopsy (78 samples total)**				
KRAS 12	27	2 × c.34G>A 1 × c.34G>T 12 × c.35G>A 2 × c.35G>C 10 × c.35G>T	5	1 × c.34G>C (5%) 2 × c.35G>A (6%, 8%) 2 × c.35G>T (5%, 6%)
KRAS 13	3	3 × c.38G>A	6	6 × c.38G>A (5%, 5%, 7%, 7%, 8%, 12%)
KRAS 61	0	—	0	—
KRAS 146	5	5 × c.436G>A	4	4 × c.436G>A (5%, 5%, 6%, 9%)
NRAS 12/13	2	c.35G>A c.37G>C	0	—
NRAS 61	1	c.181C>A	0	—
BRAF	3	NA	0	—
PIK 542	5	5 × c.1624G>A	1	1 × c.1624G>A (5%)
PIK 545/546	5	3 × c.1633G>A 1 × c.1636C>A 1 × c.1637A>C	3	2 × c.1633G>A (5%, 10%) 1 × c.1636C>A (5%)
PIK 1047	1	c.3140A>G	2	c.3139C>T (6%) c.3140A>G (7%)
Total	52		21	
Number of patients with RAS (KRAS or NRAS) mutation by PS 37 (47%)				
Number of patients with RAS (KRAS or NRAS) mutation by PS or NGS 44 (56%)				
Number of patients with EGFR pathway mutation (KRAS, NRAS, BRAF or PIK3CA) by PS 45 (58%)				
Number of patients with EGFR pathway mutation (KRAS, NRAS, BRAF or PIK3CA) by PS or NGS 50 (64%)				
**Resection (54 samples total)**				
KRAS 12	24	1 × c.34G>A 2 × c.34G>T 13 × c.35G>A 1 × c.35G>C 7 × c.35G>T	3	2 × c.34G>C (14%, 25%) c.35G>C (7%)
KRAS 13	3	3 × c.38G>A	0	—
KRAS 61	0	—	0	—
KRAS 146	2	2 × c.436G>A	3	3 × c.436G>A (5%, 5%, 33%)^a^
NRAS 12/13	1	c.37G>C	0	—
NRAS 61	1	c.181C>A	0	—
BRAF	0	—	0	—
PIK 542	3	3 × c.1624G>A	1	c.1624G>A (5%)
PIK 545/546	0	—	1	c.1633G>A (10%)
PIK 1047	1	c.3140A>G	0	—
Total	35		8	
Number of patients with RAS (KRAS or NRAS) mutation by PS 31 (57%)				
Number of patients with RAS (KRAS or NRAS) mutation by PS or NGS 33 (61%)				
Number of patients with EGFR pathway mutation (KRAS, NRAS, BRAF or PIK3CA) by PS 32 (59%)				
Number of patients with EGFR pathway mutation (KRAS, NRAS, BRAF or PIK3CA) by PS or NGS 33 (61%)				

Abbreviations: NA=not applicable; NGS=next generation sequencing; PS=pyrosequencing.

a One sample that was resection KRAS 146 mutated on PS was non-mutated on NGS.

**Table 5:** Mutation data for the 52 matched samples using mutations identified on either PS or NGS

**Pre-treatment biopsy mutation details**	**Post-resection specimen mutation details**	**Note/description**
Biopsy and resection both non-mutated (*n*=12)		
NA	NA	NA
Biopsy and resection have matching mutations (*n*=16)		
KRAS 12	KRAS 12	× 10 patients
KRAS 146	KRAS 146	× 3 patients
NRAS 12/13	NRAS 12/13	× 1 patient
PIK 542	PIK 542	× 1 patient
KRAS 13, PIK 542	KRAS 13, PIK 542	× 1 patient
Discrepant results between biopsy and resection		
EGFR pathway mutation gain between biopsy and resection (*n*=6)		
No mutation	KRAS 12 (25%)^a^	No mutation to one mutation
No mutation	KRAS 12 (27%)	No mutation to one mutation
No mutation	KRAS 12 (31%)	No mutation to one mutation
No mutation	KRAS 12 (13%), KRAS 146 (5%)	No mutation to two mutations
No mutation	KRAS 12 (14%); KRAS 12 (24%), KRAS 146 (33%)	No mutation to three mutations
KRAS 12 (15%)	KRAS 12 (18%), PIK 542 (24%)	One mutation to two mutations
EGFR pathway mutation loss between biopsy and resection (*n*=14)		
KRAS 12 (27%)	No mutation	One mutation to no mutation
KRAS 13 (12%)	No mutation	One mutation to no mutation
KRAS13 (41%)	No mutation	One mutation to no mutation
KRAS 12 (6%)	No mutation	One mutation to no mutation
KRAS 13 (27%)	No mutation	One mutation to no mutation
PIK 546 (5%)	No mutation	One mutation to no mutation
KRAS 146 (23%)	No mutation	One mutation to no mutation
KRAS 146 (5%), PIK 1047 (7%)	No mutation	Two mutations to no mutation
KRAS 12 (33%), KRAS 13 (5%)	KRAS 12 (43%)	Two mutations to one mutation
KRAS 12 (36%), PIK 542 (27%)	KRAS 12 (38%)	Two mutations to one mutation
KRAS 13 (41%), PIK 545 (40%)	KRAS 13 (25%)	Two mutations to one mutation
KRAS 12 (26%), KRAS 13 (7%)	KRAS12 (31%)	Two mutations to one mutation
KRAS 146 (6%), NRAS 61 (17%)	NRAS 61 (27%)	Two mutations to one mutation
KRAS 12 (9%), KRAS 13 (5%), PIK 545 (10%)	KRAS 12 (14%), PIK 545 (10%)	Three mutations to two mutations
EGFR pathway mutation loss and gain between biopsy and resection (*n*=4)		
KRAS 12 c.35G>A (29%)	KRAS12 c.34G>T (40%)	One mutation to non-mutated plus gained one new mutation
KRAS 146 (33%), PIK 545 (37%)	KRAS 12 (24%)	Two mutations to non-mutated plus gained one new mutation
KRAS 146 (9%), PIK 542 (5%), PIK545 (5%)	KRAS 12 (51%), PIK 542 (5%)	Three mutations to one mutation plus gained one new mutation
KRAS 146 (5%), PIK 1047 (6%), PIK1047 (29%)	KRAS 12 (34%), PIK 1047 (19%)	Three mutations to one mutation plus gained one new mutation

Abbreviations: EGFR= epidermal growth factor receptor; NA=not applicable; NGS=next generation sequencing; PS=pyrosequencing.

a The number in brackets is the amount of mutated DNA as a percentage of the total DNA present.

**Table 6:** Summary of the influence of RAS status (assessed by NGS) on histology and survival

	**Biopsy** ***N*****=78**			**Either biopsy or resection** ***N*****=80**		
	**RAS wild-type** ***N*****=34**	**RAS mutated** ***N*****=44**	***P*****-value**^a^	**RAS wild-type** ***N*****=29**	**RAS mutated** ***N*****=51**	***P*****-value**^a^
R0	25 (74%)	39 (89%)		22 (76%)	44 (88%)	
R1-2^b^	6 (18%)	3 (7%)	0.16	4 (14%)	5 (10%)	0.71
Did not have surgery^c^	3 (9%)	2 (5%)		3 (10%)	2 (2%)	
ECPR^d^	14 (41%)	10 (23%)		14 (48%)	10 (20%)	
Non-ECPR	20 (59%)	33 (75%)	0.090	15 (52%)	40 (78%)	0.008
Did not have surgery and no CCR^e^	0 (0%)	1 (2%)		0 (0%)	1 (2%)	
Progression-free survival	HR 0.53 (95% CI: 0.23 to 1.22)		0.137	HR 0.44 (95% CI: 0.18 to 1.10)		0.079
Overall survival	HR: 0.32 (95% CI: 0.09 to 1.14)		0.079	HR: 0.23 (95% CI: 0.05 to 1.03)		0.055

Abbreviations: CCR=clinical complete response; CI=confidence interval; ECPR=excellent clinical or pathological response; HR=hazard ratio; NGS=next generation sequencing; OS=overall survival; PFS=progression-free survival.

a When appropriate, Chi-square tests or Fisher’s exact test used for resection and ECPR status; log rank test used for PFS and OS.

b One patient was considered an R2 resection.

c Patients who did not have surgery were not included in the Fisher’s exact test.

d Four patients with ECPR had complete clinical responses and were managed expectantly without resection: three were biopsy KRAS/NRAS non-mutated and one was biopsy mutated.

e The RAS mutated patient who did not have surgery and did not have complete clinical response was not included in the Chi-square test analysis.
