# Supplementary material for: Preoperative chemoradiation with capecitabine, irinotecan and cetuximab in rectal cancer: significance of pre-treatment and post-resection RAS mutations
Source: Br J Cancer. 2017 Aug 31;117(9):1286–94. doi: 10.1038/bjc.2017.294 (PMC5672930; doi:10.1038/bjc.2017.294)
Supplement: Supplementary Information [file bjc2017294x1.doc]

**EXCITE Supplementary online material: Text**

**Methodology for next-generation sequencing**

**DNA extraction**

Blocks received were sectioned at 5µm and stained with haematoxylin and eosin, in order to determine the amount and area of tumour in each. Blocks were placed on ice. Ten 5µm sections were cut from each block, floated on a water bath at 45**°**C and put onto SuperFrost slides. Slides were put on a hotplate at 65°C, then sections were de-waxed and rehydrated by incubation in xylene, graded ethanols and deionized water.

Appropriate areas of tumour were macrodissected from the sections and DNA extracted using the protocol for extraction from formalin-fixed paraffin-embedded (FFPE) tissue with the Qiagen QiAmp DNA Micro Kit. Briefly, tissue was digested overnight in 180µl buffer ATL plus 20µl proteinase K in a hot block set at 56°C. To each sample was then added 200µl buffer AL, 200µl 100% ethanol, the sample was vortexed for 15 seconds and then left to incubate at room temperature for 5 minutes.

Each sample was then run through a MinElute column at 8000rpm for one minute. Two wash buffers were then added to the column in turn and run through the column at 8000rpm for one minute. The columns were then dried to remove excess ethanol by centrifuging at 14000rpm for 3 mins. DNA was then eluted by adding 30µl elution buffer to each column and centrifuging at 14000rpm for one minute. DNA was then quantified using the NanoDrop 1000 Spectrophotometer.

**Polymerase Chain Reaction (PCR)**

The PCR was set up as follows;

| **Reagent** | **Volume per tube/well (µl)** |
| --- | --- |
| Phusion Hot Star Flex MM (2x) | 12.5 |
| DMSO | 0.75 |
| Fwd Primer (25uM) | 0.5 |
| Rev Primer (25uM) | 0.5 |
| dH_2_0 | 8.75 |
| DNA (10ng/ul nanodrop result) | 2 |
| TOTAL | 25 |

| **Temperature (^o^C)** | **Time (seconds)** | **Cycles** |
| --- | --- | --- |
| 98 | 30 | 1 |
| 98 | 10 | 35 cycles |
| 55 | 20 |
| 72 | 20 |
| 72 | 5 minutes | 1 |
| 4 | Infinite hold | 1 |

**PCR product purification**

Each PCR product from each case was pooled. This was then purified using the Qiagen QIAquick PCR purification kit. Briefly, five volumes of buffer PB was added to each pool. This was transferred to a MinElute column and centrifuged at 13000rpm for one minute and the flowthrough discarded. 750µl buffer PE was then added to each column, which was then centrifuged at 13000rpm for one minute, flowthrough discarded. Residual ethanol was then removed by centrifugation for a further one minute then DNA was eluted by adding 10µl buffer EB to the column, incubation at room temperature for one minute then centrifugation at 13000rpm for one minute.

**Library prep**

Library prep used reagents from the NEBNext Ultra DNA Library Prep Kit from New England Biolabs. 45.5µl TE buffer was added to the purified PCR products. The end repair mastermix was made up as follows:

| **Reagent** | **Volume per tube/well (µl)** |
| --- | --- |
| End Prep Enzyme Mix | 3.0 |
| End Repair Reaction Buffer (10x) | 6.5 |
| TOTAL | 9.5 |

End repair mastermix was added to the purified PCR products and mixed to give a final volume of 65µl. This was incubated in a heat block or thermocycler as follows:

| **Temperature (^o^C)** | **Time (minutes)** |
| --- | --- |
| 20 | 30 |
| 65 | 30 |
| 4 | Infinite hold |

Adapter ligation mastermix was made up as follows:

| **Reagent** | **Volume per tube/well (µl)** |
| --- | --- |
| Blunt/TA Ligase mastermix | 15 |
| NEBNext Adaptor for Illumina | 2.5 |
| Ligation Enhancer | 1.0 |
| TOTAL | 18.5 |

18.5ul was then added to each sample and mixed to give a final volume of 83.5ul. This was incubated in a heat block or thermocycler for 15 min at 20°C. The sample was spun briefly, then 3µl USER enzyme (NEBNext Ultra kit) was added and mixed for a final volume of 86.5µl. This was incubated for 15 min at 37°C.

**Bead clean-up 1**

Each reaction mix was transferred to a 1.5ml Eppendorf tube. Magnetic beads were added to each reaction, totalling 0.65 x volume of reaction mix (56.2µl in this instance). These were incubated at room temperature for 5 min and briefly centrifuged. Each Eppendorf tube was then transferred to a magnetic rack and left to separate for 5 min at room temperature. The supernatant was discarded and 200µl 80% ethanol was added to each Eppendorf while on the magnetic rack. This was left to incubate for 30 secs then supernatant was removed. Beads were washed with ethanol a further 2 times and left to air-dry for 10 minutes to remove any residual ethanol. The tubes were taken out of the rack and 28µl buffer EB was added to each and the beads resuspended. The mixes were each spun briefly, and put back onto the magnetic rack for 5 min in order for the beads to separate out again. 23µl of the resulting supernatant was then transferred to a new 0.2ml strip tube.

**Enrichment PCR**

Enrichment PCR mastermix was made up as follows:

| **Reagent** | **Volume per tube/well (µl)** |
| --- | --- |
| NEBNext High Fidelity 2X PCR Master Mix | 25 |
| Universal PCR Primer | 1 |
| TOTAL | 26 |
|  |  |
| Cleaned sample from previous step | 23 |
| Unique barcode (record which for each sample) | 1 |
| TOTAL | 50 |

Mastermix and unique barcode were added to each 0.2ml strip tube for a final volume of 50µl.

Each sample was run under the following thermocycling conditions:

| **Step** | **Temperature (^o^C)** | **Time** | **Cycles** |
| --- | --- | --- | --- |
| Initial Denaturation | 98 | 30 sec | 1 |
| Denaturation | 98 | 10 sec | 12 |
| Annealing | 65 | 30 sec |
| Extension | 72 | 30 sec |
| Final Extension | 72 | 5 minutes | 1 |
| Hold | 4 | ∞ |  |

**Bead clean-up 2**

Each PCR reaction was transferred to a new 1.5ml eppendorf tube. Magnetic beads were added to each reaction, totalling 0.65 x volume of reaction mix (32.5µl in this instance). These were incubated at room temperature for 5 min and briefly centrifuged. Each eppendorf tube was then transferred to a magnetic rack and left to separate for 5 min at room temperature. The supernatant was discarded and 200µl 80% ethanol was added to each eppendorf while on the magnetic rack. This was left to incubate for 30 secs then supernatant was removed. Beads were washed with ethanol once more and left to air-dry for 10 minutes to remove any residual ethanol. The tubes were taken out of the rack and 33µl buffer EB was added to each, the beads resuspended. The mixes were each spun briefly, and put back onto the magnetic rack for 5 min in order for the beads to separate out again. 28µl of the resulting supernatant was then transferred to a new 1.5ml eppendorf tube.

**Quantitation of libraries**

Each library was quantitated on the Agilent 2200 TapeStation: 1ul DNA plus 3µl sample buffer. 20ng of each library was pooled and sequenced on an Illumina MiSeq instrument, using a 2x 150bp run.
