# Supplementary material for: Preoperative chemoradiation with capecitabine, irinotecan and cetuximab in rectal cancer: significance of pre-treatment and post-resection RAS mutations
Source: Br J Cancer. 2017 Aug 31;117(9):1286–94. doi: 10.1038/bjc.2017.294 (PMC5672930; doi:10.1038/bjc.2017.294)
Supplement: Supplementary Figure 1 [file bjc2017294x2.doc]

**EXCITE Supplementary online material Figures 1a and 1b.**

**Online-only Figure 1a.** Progression-free survival for patients with ECPR versus non-ECPR

36 month PFS: ECPR: 95% (95% CI: 74-99%); Non-ECPR: 54% (95% CI: 39-66%).

**Online-only Figure 1b.** Overall survival for patients with ECPR versus non-ECPR

36 month OS: ECPR: 95% (95% CI:72-99%); Non-ECPR: 73% (95% CI: 58-83%).
