# Supplementary material for: Preoperative chemoradiation with capecitabine, irinotecan and cetuximab in rectal cancer: significance of pre-treatment and post-resection RAS mutations
Source: Br J Cancer. 2017 Aug 31;117(9):1286–94. doi: 10.1038/bjc.2017.294 (PMC5672930; doi:10.1038/bjc.2017.294)
Supplement: Supplementary Tables [file bjc2017294x3.doc]

**EXCITE Supplementary Online Material, Tables**

**Online Material Table 1. EXCITE treatment compliance**

**Radiotherapy**

Full dose (45 Gy) received without delay as per protocol 47 (57%)

Full dose (45 Gy) received with delay due to adverse events 29(35%)

Dose reduction 4 (5%)

Did not start 2 (2%)

Median dose received in Gy (IQR) 45 (45-45)

**Irinotecan**

Full dose received (240 mg/m^2^) without delay 46 (56%)

Full dose received (240 mg/m^2^) with delay 10 (12%)

Dose reduction 24 (29%)

Did not start 2 (2%)

Number of cycles given

0 2 (2%)

1 0

2 5 (6%)

3 18 (22%)

4 57 (70%)

Median dose received (mg/m^2^) 238 (180-242)

**Cetuximab**

Full dose received (1650 mg/m^2^) without delay 51 (62%)

Full dose received (1650 mg/m^2^) with delay 9 (11%)

Dose reduction 21 (26%)

Did not start 1 (1%)

Number of cycles given

0 1 (1%)

1 1 (1%)

2 0 (0%)

3 2 (2%)

4 2 (2%)

5 14 (17%)

6 62 (76%)

Median dose received (mg/m^2^) 1650 (1548-1657)

**Capecitabine**

| Full dose received without delay | 35 (43%) | |  |
| --- | --- | --- | --- |
| *Alteration to capecitabine due to: | 45 (55%) | |  |
| treatment not taken | | 8 (10%) | |
| treatment reduction | | 12 (15%) | |
| treatment delayed | | 4 (5%) | |
| treatment not taken and reduced | | 9 (11%) | |
| treatment not taken and delayed | | 1 (1%) | |
| treatment reduction and delayed | | 9 (11%) | |
| treatment not taken, reduced and delayed | | 2 (2%) | |
| Did not start Capecitabine | 2 (2%) | |  |

*In addition to the central record of the dose of capecitabine prescribed, patients kept a weekly record of prescribed tablets that were not taken

**Online Material Table 2. Details of surgery in the 76 patients undergoing resection^1^, together with post operative complications within 30 days of surgery**

| **Type of surgery** | **Number (%)** |
| --- | --- |
| Abdominoperineal excision | 38 (50%) |
| Anterior resection | 36 (47%) |
| Hartmann’s procedure | 2 (3%) |
|  |  |
| **Complications within 30 days of surgery** |  |
| Anastomotic dehiscence | 3 (4%) |
| Perineal wound dehiscence | 7 (9%) |
| Haemorrhage within the operative field necessitating return to theatre | 1 (1%) |
| Wound infection | 12 (16%) |
| Pelvic infection | 4 (5%) |
| Serious infection elsewhere | 7 (9%) |
| Peritonitis | 2 |
| Pneumonia | 1 |
| Presacral collection | 1 |
| Subphrenic | 1 |
| Cannula site | 1 |
| Urinary sepsis | 1 |
| Re-catheterisation | 12 (16%) |
| Venous thromboembolic event | 1 (1%) |
| Myocardial infarction | 0 (0%) |
| Cerebrovascular accident | 0 (0%) |
| Ventilation required for >24 hours | 0 (0%) |
| Acute respiratory distress syndrome | 0 (0%) |
| Re-admission after discharge | 13 (17%) |
| Death within 30 days of operation | 1 (1%) |
| Other ^2^ | 9 (12%) |
|  |  |
| Any (of the above) post-surgical complications | 33 (43%) |
|  |  |
| **Time spent on ITU/HDU post-op (days)** |  |
| 0 | 32 (42%) |
| 1 | 14 (18%) |
| 2-5 | 15 (20%) |
| 6-10 | 2 (3%) |
| Missing | 13 (17%) |
| Median in days (IQR) | 0.5 (0 to 2) |
|  |  |
| **Total time as in-patient, post-op (days)** |  |
| 0 | 1 (1%) |
| 1-10 | 45 (59%) |
| 11-20 | 14 (18%) |
| 21-30 | 5 (7%) |
| 31-40 | 2 (3%) |
| Missing | 9 (12%) |
| Median in days (IQR) | 8 (5.5 to 12) |
|  |  |
|  | |
| ^1^In 4 patients a ‘wait and watch’ approach was adopted by the treating team because of a complete clinical response to CRT.  ^2^ Nine patients had 15 grade 3-5 “other” surgical complications post-surgery: One patient had two grade 5 events: ileus and aspiration (and grade 4 vomiting). Two patients had a maximum grade 4 (small bowel obstruction; bleeding associated with surgery). The other six patients had a maximum of grade 3 - abdominal pain (3); DVT (1); type 2 respiratory failure (1); shortness of breath (1); chest infection (1); rectal/pelvic pain (1); oedema (1); low magnesium (1). | |

**Online Material Table 3. Number of EGFR pathway mutations per sample (including detail of samples containing multiple mutations) in biopsy and resection specimen**

| **BIOPSY** | | |
| --- | --- | --- |
|  | **Number of samples containing indicated number of EGFR pathway mutations by PS/NGS*** | **Detail (percentage of mutant DNA)** |
| No mutation | 28 (36%) |  |
| Single mutation | 33 (42%) | - |
| Double mutation | 12 (15%) | KRAS 12 c.35G>A (35%)  & KRAS 12 c.35G>T (16.6%) |
|  |  | KRAS 12 (33%) & KRAS 13 (5%) |
|  |  | KRAS 12 (26%) & KRAS 13 (7%) |
|  |  | KRAS 12 (22%) & PIK 545/6 (26%) |
|  |  | KRAS 12 (36%) & PIK 542 (27%) |
|  |  | KRAS 12 (8%) & BRAF (22%) |
|  |  | KRAS 13 (41%) & PIK 545/6 (40%) |
|  |  | KRAS 13 (7%) & PIK 542 (9%) |
|  |  | KRAS 146 (6%) & NRAS 61 (17%) |
|  |  | KRAS 146 (33%) & PIK 545/6 c.(37%) |
|  |  | KRAS 146 (5%) & PIK 1047 (7%) |
|  |  | BRAF (30%) & PIK 545/6 (25%) |
| Triple mutation | 4 (5%) | KRAS 12 (28%) & KRAS 13 (8%) & PIK 542 (28%) |
|  |  | KRAS 12 (9%) & KRAS 13 (5%) & PIK 545/6 (10%) |
|  |  | KRAS 146 (9%) & PIK 542 (5%) & PIK 545/6 (5%) |
|  |  | KRAS 146 (5%) & PIK 1047 (6%) & PIK 1047 (29%) |
| Quadruple mutation | 1 (1%) | KRAS 12 (5%) & KRAS 12 (6%) & KRAS 12 (5%) & NRAS 12/13 c.35G>A (24%) |
| Total | 78 (100%) |  |
| **RESECTION** | | |
|  | **Number of samples containing indicated number of mutations by PS/NGS**** | **Detail (percentage of mutant DNA)** |
| No mutation | 20 (37%) | - |
| Single mutation | 26 (48%) |  |
| Double mutation | 7 (13%) | KRAS 12 (25%) & KRAS 12 (7%) |
|  |  | KRAS 12 c.35G>T (13%) & KRAS 146 c.436G>A (5%) |
|  |  | KRAS 12 (18%) & PIK 542 (24%) |
|  |  | KRAS 12 (51%) & PIK 542 (5%) |
|  |  | KRAS 12 (14%) & PIK 545/6 (10%) |
|  |  | KRAS 12 (34%) & PIK 1047 (19%) |
|  |  | KRAS 13 (35%) & PIK 542 (26%) |
| Triple mutation | 1 (2%) | KRAS 12 (14%) & KRAS 12 (24%) & KRAS 146 (33%) |
| Total | 54 |  |

NA: not applicable; PS: pyrosequencing; NGS: next generation sequencing

*One sample did not have enough DNA to run matched NGS (KRAS 12 mutant on PS)

**Four samples did not have enough DNA to run matched NGS (two non-mutated, one KRAS 12 mutated and one KRAS 13 mutated on PS)
